# Trigger-Responsive Gene Transporters for Anticancer Therapy

**DOI:** 10.3390/nano7060120

**Published:** 2017-05-26

**Authors:** Santhosh Kalash Rajendrakumar, Saji Uthaman, Chong Su Cho, In-Kyu Park

**Affiliations:** 1 Department of Biomedical Science and BK21 PLUS Center for Creative Biomedical Scientists at Chonnam National University, Chonnam National University Medical School, Gwangju 61469, Korea; kalash1288@gmail.com (S.K.R.); sajiuthaman@gmail.com (S.U.); 2 Department of Agricultural Biotechnology and Research Institute for Agriculture and Life Sciences, Seoul National University, Seoul 08826, Korea

**Keywords:** cationic polymer, gene delivery, non-viral, anti-cancer, trigger-responsive, photothermal, glutathione, ultrasound, magnetic field

## Abstract

In the current era of gene delivery, trigger-responsive nanoparticles for the delivery of exogenous nucleic acids, such as plasmid DNA (pDNA), mRNA, siRNAs, and miRNAs, to cancer cells have attracted considerable interest. The cationic gene transporters commonly used are typically in the form of polyplexes, lipoplexes or mixtures of both, and their gene transfer efficiency in cancer cells depends on several factors, such as cell binding, intracellular trafficking, buffering capacity for endosomal escape, DNA unpacking, nuclear transportation, cell viability, and DNA protection against nucleases. Some of these factors influence other factors adversely, and therefore, it is of critical importance that these factors are balanced. Recently, with the advancements in contemporary tools and techniques, trigger-responsive nanoparticles with the potential to overcome their intrinsic drawbacks have been developed. This review summarizes the mechanisms and limitations of cationic gene transporters. In addition, it covers various triggers, such as light, enzymes, magnetic fields, and ultrasound (US), used to enhance the gene transfer efficiency of trigger-responsive gene transporters in cancer cells. Furthermore, the challenges associated with and future directions in developing trigger-responsive gene transporters for anticancer therapy are discussed briefly.

## 1. Introduction

**Figure 1:**
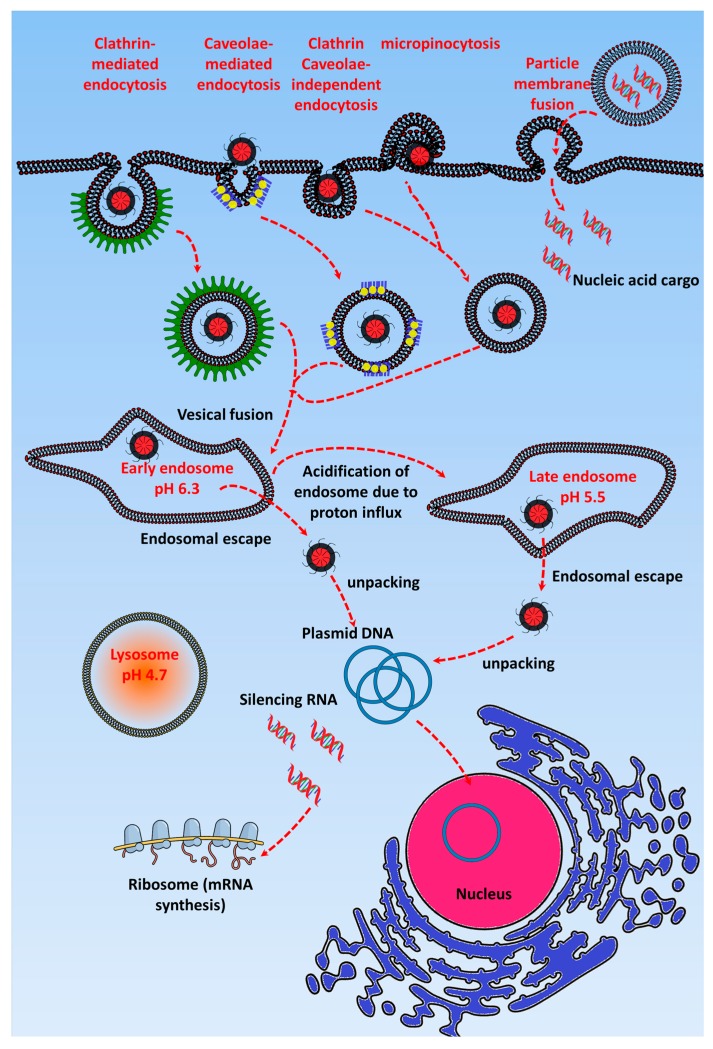
Schematic representation of gene transporter delivery into cancer cells: Gene transporters are uptaken by the cells via various pathways and then are accumulated in the early endosome (pH 6.3). Later, they are transported to the late endosome before entering the lysosome. However, via the proton sponge effect, they escape from the late endosome and later release their nucleic acid cargo (silencing RNA or plasmid DNA). The purple, orange, and green stars indicate the rate-limiting barriers, and the red star indicates the fate of an ineffective gene transporter in the lysosome. This scheme was drawn with help of Inkscape and www.mindthegraph.com icons.

Because of the discovery and development of new therapeutic genes and their delivery agents, cancer gene therapy research has been advancing at rapid pace, although the key challenges in the field remain immunogenicity, off-target toxicity, and low sustaining efficacy in the blood stream [1]. Naked nucleic acids in their native state are inefficiently delivered to cells, and they therefore must be protected for safe delivery into target cells to obtain efficient transgene expression or target protein silencing in cancer cells [2]. Several notable drawbacks associated with delivering naked nucleic acids are poor internalization by passive diffusion, which is likely due to their size, negative charge, hydrophilicity, and susceptibility to nuclease enzyme attack [2]. Over the past few decades, non-viral gene delivery methods, in which gene carriers are synthetic or natural materials, have become an alternative to viral gene delivery. Nucleic acids such as plasmid DNA, siRNA, miRNA, and oligonucleotides can be either loaded into or conjugated onto cationic or non-cationic gene transporters (GTs) by one of the following methods: (a) conjugation of nucleic acids onto a nanoparticle surface via various functional groups; (b) encapsulation of nucleic acids inside the hydrophilic core of a nanoparticle; or (c) complexation between DNA and a nanoparticle via electrostatic interaction. Depending on the delivery strategies, nucleic acids are delivered and released into the cytoplasm or nucleus by different mechanisms. The intracellular uptake of nucleic acids delivered with GTs is mostly mediated by caveolae- or clathrin-dependent endocytic pathways, whereas nucleic acid release from the endosome can be triggered either by an external source, such as light, a magnetic field, or ultrasound (US), or an internal source, such as enzymes or a redox environment. Nucleic acids can form interpolyelectrolyte complexes with cationic polymers, and this interaction creates nanosize aggregates that possess a positive surface charge [3]. Cationic GTs, such as polyethylenimine (PEI), polylysine (PLL), polyamidoamine (PAMAM) dendrimers, and chitosan, and lipids, such as 2,3-dioleyloxy-*N*-[2-(sperminecarboxamido)ethyl]-*N*,*N*-dimethyl-1-propanaminium trifluoroacetate (DOSPA), *N*-[1-(2,3-dioleoyloxy)propel]-*N*,*N*,*N*-trimethylammonium (DOTMA), 1,2-dioleoyl-3-trimethylammonium-propane (DOTAP) are widely used for gene delivery [4,5]. Cationic polymers have characteristic properties such as high charge density and buffering capacity that enable them for nucleic acid condensation and endosomal escape inside cells, whereas liposomes or lipid-based gene transporters containing encapsulated or condensed nucleic acids deliver their cargo by fusing with the cell membrane, releasing it into the cytoplasm (as shown in Figure 1). Non-cationic GTs, such as gold nanoparticles, super paramagnetic iron oxide nanoparticle (SPION), and lipopolythioureas, have also been used for gene delivery, but nucleic acids have to be either directly conjugated onto the surface of the particle or complexed to the surface, which is modified with cationic polymers or lipids via electrostatic interaction [6,7,8,9,10]. The initial key factor that determines the gene transfer efficiency of cationic or non-cationic GTs is their ability to condense nucleic acids and release them efficiently into the cytoplasm.

Generally, the condensation of DNA by a cationic GT, which act as a condensate, involves electrostatic interaction and hydration forces between the phosphate group of DNA and the amine group of the GT [11]. The GT can condense DNA into different morphologies, such as toroids, rods, spheres, ovals, disks, and flower-like aggregates, depending on the condensate’s surface charge and shape [12]. The complete compact packing of DNA by GTs sometimes results in reduced gene transfection efficiency in vitro, and therefore, modification of the GT is required in order to unpack the DNA in the cytoplasm for gene transcription [13]. Incorporating enzyme-, pH-, heat- or hypoxia-sensitive polymers or linkers into to the GT has been shown to improve their efficiency in releasing the condensed nucleic acid, but the modification of GTs needs to be done in a controlled manner and allow some space for the DNA to condense. For example, PEGylation of liposomes or polymeric GTs is done in order to provide serum stability and prevent aggregation in the blood stream, but at the same time, it hinders DNA condensation [14,15]. Therefore, it is necessary to achieve modification without affecting the key factors responsible for efficient gene transfection. 

Another important factor to be considered regarding GTs is tumor specific gene release, where the GT has to specifically bind to cancer cells, be internalized, and release the nucleic acid cargo for later transgene expression or gene silencing. However, for efficient gene release, a trigger is required for the GT to precisely enter the cancer cell and release the gene into the cytoplasm for either transgene expression or gene silencing. The trigger can be either intrinsic, such as an enzyme or pH, or extrinsic, such as light or a magnetic field. Tumor tissues are distinctly separate from healthy tissues based on various factors, such as pH, extracellular and intracellular enzymes, and permeability of blood vessels, and accordingly, GTs can be developed to respond to these factors for tumor-specific gene release. In addition, physical triggers such as light, US, magnetic fields, and electrical fields can also be applied to facilitate a GT to be internalized by cancer cells and release its cargo. Trigger-responsive GTs demonstrate “on demand” gene release, thus avoiding off-target healthy cells and efficiently releasing nucleic acids in the cytoplasm of cancer cells. Thus, studies have shown that decreased side effects and better therapeutic outcomes can be achieved both in vitro and in vivo [16]. In this review, we summarize the triggers, such as enzymes, light, US and magnetic fields, and the GTs that respond to these triggers.

## 2. Mechanism for Effective Release of Exogenous Nucleic Acids by GT in Cancer Cells

After reaching the tumor environment, GTs are internalized into the cells via either endocytic pathways such as clathrin or caveolae or non-endocytic pathways such as particle membrane fusion. The internalization pathway of GTs depends on their characteristics, such as surface charge, shape, and surface conjugated ligand. These internalization pathways also determine the transfection efficiency of GTs, and it was shown by Douglas et al. that chitosan-alginate DNA complexes up taken by non-endocrine cell linesvia the caveolae pathway are entrapped in caveosomes, whereas in the case of complexes taken up via the clathrin-mediated pathway in the 293T and COS7 cell lines, endosomal escape eventually facilitates high transfection efficiency [17]. The internalization route also depends on the type, size, and formulation of GT, along with the specific target cells to which the nucleic acid cargo is delivered [18].

More than nucleic acids been internalized via endocytic pathways, non-endocytic pathway routes provide high accumulation in the nucleus. The most common technique such as electroporation or nucleofection has higher gene transfection efficiency because the nucleic acids are delivered directly into the cytosol or nucleus through the cell membrane pores created by a short electrical impulse [19,20,21]. GTs such as cationic lipoplexes follow the cell membrane fusion route to deliver nucleic acids, although it depends on the size i.e., large size liposomes containing nucleic acids fuse with the cell membrane, whereas small size liposomes follow the endocytic pathways [22]. Apart from these, cell penetrating peptides (CPPs) assist in delivering nucleic acids by translocating into the cells via direct membrane translocation, although most studies suggest that CPP conjugated GTs follows the endocytic pathway [23,24]. CPP containing cargo being directly translocation or internalized by endocytosis depends on the size of the cargo. Mishra et al. showed that human immunodeficiency virus (HIV) transactivator (TAT) peptide conjugated to a small cargo can easily translocate through the membrane via forming a membrane pore, but if the cargo is of a few nanometer size more, then it will bind to the cell membrane and eventually get internalized via the endocytic pathway [25]. In order to develop GTs following non-endocytic pathway, certain criteria have to be followed such as large size, amphiphilicity, and anionic nature [26,27,28,29]. Above all, it is quite necessary to study the non-endocytic pathway in more detail and it is possible by using computational tools, fluorescent probes, and cut through techniques to know the in depth nature of the cell membrane penetration of GTs.

Figure 1 shows that after GTs enter the cells via endocytic pathways, they are initially accumulated in the early endosome (pH 7.4). Here, cationic GTs in particular act as a “proton sponge” and cause endosomal maturation, where pH acidification occurs due to H+ATPase activity [30,31,32,33,34]. The “proton sponge effect” is the generally accepted mechanism for endosomal escape, and the hypothesis, which was proposed by Behr in 1997, states that the unprotonated amines of cationic GTs can absorb the proton influx in the endosome/lysosome, resulting in an increased influx of Cl^−^ ions and water [35]. The osmotic swelling of the cationic polymer because of repulsion between the protonated amine groups causes rupture in the lysosomal membrane and leads to subsequent release of its contents into the cytoplasm [36]. Many have observed a reduction of the pH in the lysosome after the uptake of cationic polymers, and the cause of this pH reduction is the buffering capacity of the cationic polymers. Using pH probes, studies have been done to assess the pH reduction range in cationic nanoparticle-containing lysosomes and found it to be around pH 5.5. However, some researchers have contradicted this concept by experimentally proving that the proton sponge effect does not change the pH of the lysosome [37,38]. Still other studies have shown that the buffering of the endosome by cationic polymers is responsible for the endosomal escape of the polyplexes [35,36]. Singh et al. demonstrated that higher cellular uptake of polyplexes will not always result in higher transfection efficiency if the polymer has the optimal buffering capacity to release the gene from the endosome [39]. Most GTs escape from the late endosome, but some, such as cell penetrating peptide-based GTs, have been found to escape efficiently via the early endosome [34,40]. After the endosomal escape, the gene has to be released from the gene transporter and enter the nucleus via nuclear pores [41]. However, this release requires the contact of the GT with the cell membrane or vesicles, and the nuclear membrane should be permeable to facilitate the entry of the plasmid DNA, which occurs only during mitosis cell division [42]. Through the incorporation of pH-sensitive linkers that hydrolyze at the late endosomal pH of 5, the nucleic acid cargo of the GT is released in the cytoplasm [43]. For polymer-based cationic GTs, endosomal escape depends on the molecular weight of the GT; i.e., higher molecular weight provides higher buffering capacity, which leads to efficient gene release [44].

Even though GTs efficiently escape from the endosomal vesicle, they have to release their nucleic acid cargo for it to enter the nucleus via nuclear pores [41]. It was reported that nucleic acids retaining a slight positive charge from the cationic polymer can enter nuclear pores to achieve stable gene expression [45]. Developing a GT that can meet multiple requirements, including specificity, stability, and a high capacity for carrying cargos such as drugs and genes together, requires multicomplex design. Interestingly, Ashley et al. developed a GT called the protocell, which consists of a liposomal structure with an inner porous silica core loaded with epidermal growth factor receptor (EGFR), Vascular Endothelial Growth Factor Receptor-2 (VEGFR-2), and platelet-derived growth factor receptor alpha (PDGFR-α) siRNA as well as different chemotherapeutic drugs, such as cisplatin, doxorubicin, and 5-fluorouracil. The siRNA cocktail-loaded protocell alone induced 50% apoptosis in the Hep3B cell line after a 36 h incubation, but it did not affect the viability of normal hepatocytes [46]. Here, the targeting and pH-sensitive peptides also played a key role in specifically targeting the protocell toward the hepatocellular carcinoma cells.

## 3. Triggers for Gene Release in Cancer Cells Using Gene Transporters

Although cationic GTs are capable of condensing anionic nucleic acids and facilitating intracellular trafficking, restricted intracellular nucleic acid release from the endosome or GT itself is a critical roadblock to effective gene transfection [47]. Therefore, to release the nucleic acids, it is necessary to employ external triggers such as light, magnetic field, and US, along with a certain level of support from internal triggers such as protease and glutathione enzymes in the endosome/cytoplasm [48,49,50,51]. Table 1 elucidates the different triggers and its based gene transporters with their potential outcome in cancer gene therapy and also each of these triggers used for gene delivery are discussed in detail below:

### 3.1. Enzyme-Triggered Gene Release (Enz-TGR) 

**Figure 2:**
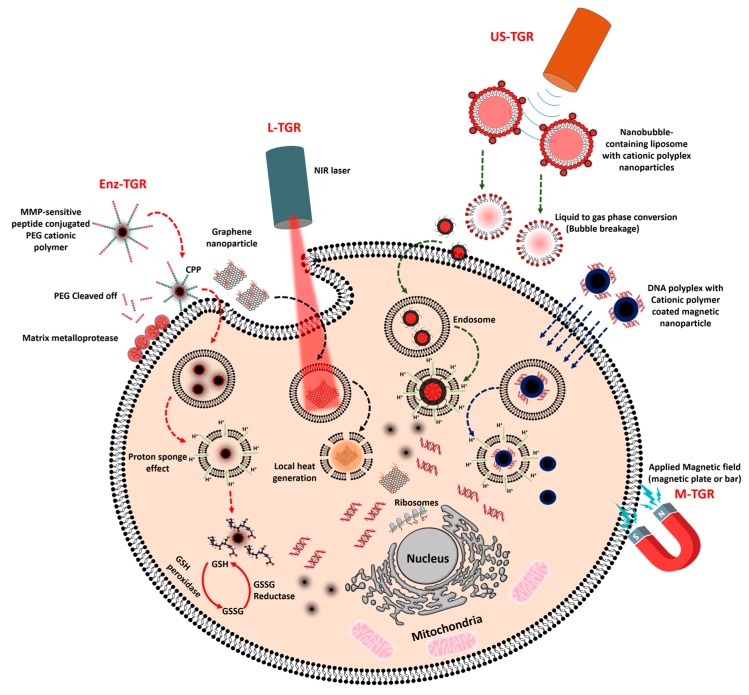
Overview of the triggered release of nucleic acids inside cells. Enz-TGR: Enzyme-triggered gene release, L-TGR: Light-triggered gene release, US-TGR: Ultrasound-triggered gene release, and M-TGR: Magnetic-triggered gene release. Scheme was drawn with the help of Inkscape and www.mindthegraph.com icons.

Enzyme-mediated gene release is mediated either by the enzymes in the extracellular environment or by the enzymes in the intracellular region. In the extracellular environment of a malignant tumor, metastasis of the cancer cells is initiated by releasing a massive amount of matrix-degrading enzymes, which creates a path for them to move on and substitute themselves into normal tissues [82], whereas in the intracellular region, glutathione is produced in large amount by the glutathione reductase enzyme (conversion of GSSG to GSH), which protects cells against reactive oxygen species (ROS). Commonly used lipoplex and polyplex approaches, where the DNA is non-covalently condensed into nanoparticles, lack in vivo efficacy and thus represent a major barrier to the translation of gene therapeutic applications to clinical trials. Therefore, certain strategies have been implemented to overcome this obstacle by developing multifunctional GTs that release their DNA cargo only when they encounter either extracellular or intracellular enzymes. Liis et al. have developed a system to deliver genes intravenously, in which a cationic cell penetrating peptide, PepFect14 (PF14), is conjugated to PEG via an MMP2-cleavable peptide linker. Thus, the gene transporter provides compact gene complexes with a shielding effect, and the cargo is released at the tumor site, where the matrix metalloproteinase enzyme concentration is higher (Figure 2) [48]. 

#### 3.1.1. Protease-Triggered Gene Release

**Figure 3:**
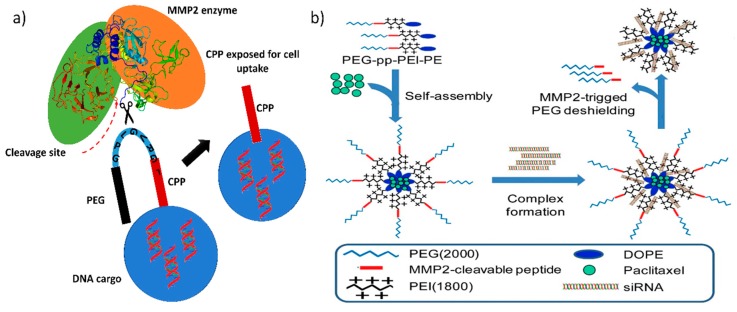
Peroxidase enzyme-triggered gene release: (**a**) Schematic diagram showing that the matrix metalloproteinase-2 (MMP2) enzyme-mediated cleavage of the linker leads to the release of Polyethylene glycol (PEG) and exposure of the cell penetrating peptide (CPP), facilitating the tumor-specific cellular uptake of DNA cargo and (**b**) drug and gene delivery strategy using an MMP2-sensitive peptide linking the PEG and polyethylenimine (PEI)-lipids. Reproduced with permission from [56]. Biomaterials, Elsevier, 2017.

Matrix metalloproteinases (MMP), a class of proteins belonging to the metzincin superfamily, are well known for their role in tumor invasion, metastasis, and angiogenesis. Generally, MMP2 and MMP9 are found in high concentrations in metastatic carcinoma, and MMP2 is responsible for breaking down the extracellular matrix to progress the cancer cells toward metastasis and neoplastic growth; thus, in almost every tumor, it is overexpressed and hence has been considered as a marker for malignancy [83]. Modern cationic polymers or liposomes have a higher uptake in cancer cells because of their high positive charge density, which attracts them to the negatively charged cell membrane. However, they mostly aggregate in the presence of serum, making cell uptake less feasible. Therefore, to keep them stable in serum, PEGylation is necessary. Conjugating PEG to either a cationic polymer or liposome increases serum stability because it helps to improve the long-term circulation of the nano gene transporters in the body However, it severely inhibits active gene transfer, particle binding to the cell surface, and endosomal release of the DNA cargo in the cytoplasm [84,85]. Hence, site-specific removal of PEG is recommended and can be achieved by conjugating PEG via protease enzyme-sensitive linkers (Figure 3a). Bruun et al. developed a lipid-based protease enzyme-sensitive GT composed of 1,2-distearoyl-sn-glycero-3-phosphoethanolamine-*N*-[amino(polyethylene glycol)-2000] (DSPE PEG_2000_) and cholesterol PEGylated cleavable lipopeptide for the delivery of siRNA across the blood-brain barrier and to glioma cells. The lipids are conjugated with PEG_2000_ via an MMP2 protease-cleavable tridecapeptide, thus providing shielding as well as site specific delivery of the siRNA cargo across the blood-brain barrier [86]. The PEGylation of cationic nanoparticle shifts their charge density toward neutral and therefore reduces their uptake in cells. The protease cleavable linker can also act as a triggered charge switch that, upon exposure to the metalloprotease enzyme, will expose the native charge of the GT or the cell penetrating peptide for specific cancer cell uptake [87]. 

Protease cleavable linkers are mostly peptide-based, but Rozema et al. used *p*-amino benzyloxy carboxyl (PABC), a protease sensitive spacer that upon exposure to MMP2 is cleaved and produces similar byproducts, such as amines and CO_2_, as protease-sensitive peptides [88].

Table 1 shows that MMP2-sensitive peptides are accompanied by cell penetrating peptide or cationic polymer, which mediates the selective exposure of the peptide or the cationic polymer near the tumor site, providing reduced off-target effects and greater availability of the therapeutic nucleic acid in the tumor than in non-cancerous tissue. Huang et al. modified dendrigraft poly-l-lysine (DGL G3) with an activatable cell penetrating peptide quenched by a pH-sensitive masking peptide and the linker between these peptides is an MMP2 sensitive peptide (*dt*ACPPD) [89]. After systemic administration, the *dt*ACPPD/DNA complexes were selectively accumulated in tumor sites via the EPR effect. Further internalization into the intratumoral cells by the CPP, exposed due to MMP2 peptide cleavage, has enabled greater GFP expression. In clinical trials, gene therapy enhances chemotherapy tolerance and enhances its therapeutic efficacy in cancer patients; therefore, strategies must be employed for delivering both genes and chemotherapy drugs in order to obtain a synergistic effect [90]. Zhu et al. constructed a MMP2-sensitive copolymer (PEG-pp-PEI-PE) that forms a self-assembled nanoparticle for tumor-targeted co-delivery of anti-survivin siRNA and paclitaxel (Figure 3b and Table 1) [56]. This type of design has proven to be target specific and has achieved high cellular internalization and enhanced synergistic antitumor activity of the co-loaded siRNA and hydrophobic drugs.

#### 3.1.2. Glutathione Enzyme-Triggered Gene Release

**Figure 4:**
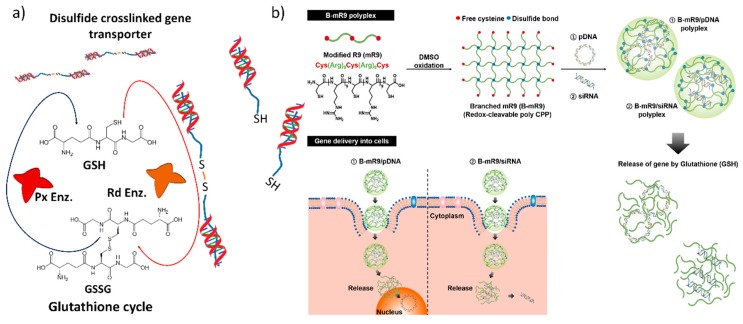
Glutathione enzyme-triggered gene release: (**a**) Schematic diagram of glutathione enzymes (glutathione cycle) involved in gene transporter disulfide bond breakage. Px Enz: Peroxidase enzyme, Rd Enz: Reductase enzyme. (**b**) Schematic illustration of the synthesis of the branched modified R9 (B-mR9) cell penetrating peptide (CPP) and construction of pDNA and siRNA polyplexes. Reproduced with permission from [97]. Journal of Controlled Release, Elsevier 2017.

Glutathione (γ-glutamyl-cysteinyl-glycine; GSH), with an intracellular concentration of ≤10 mM, is the most abundant tripeptide naturally produced by mammalian cells. GSH reacts with hydrogen peroxide as a free radical generator to form glutathione disulfide (GSSG), a reaction catalyzed by glutathione peroxidase; conversely, glutathione reductase enzyme will reduce GSSG to glutathione [91]. Under normal conditions, GSH exists mainly in its reduced form, the oxidation of which results in oxidized glutathione (GSSG), which is carried out by either direct interaction with reactive oxygen radicals (ROS) or disulfide bonds [92]. It plays an important role in antioxidant defense and regulation of cellular processes, such as DNA and protein synthesis, cell proliferation and apoptosis, cytokine production, and immune response [93]. In tumor tissues, GSH is present at millimolar (mM) concentrations inside the cells, whereas extracellular GSH is only at micromolar (μM) concentrations [94]. Hence, this differential factor provides a unique opportunity to selectively trigger gene release in cancer cells and ensure stability during systemic circulation [95,96]. A disulfide bond is a prevalent linker for reduction at tumor sites due to these having a more reducing intracellular environment than normal sites (Figure 4a). Yoo et al. synthesized branched polyarginine peptides with cysteine residues forming disulfide bonds that release VEGF siRNA in the presence of intercellular GSH, as shown in Figure 4b [97]. Introducing the disulfide bonds into the cationic polymers reduced cell toxicity and improved transfection efficiency [98,99]. Shahrouz et al. synthesized lower molecular weight PEI conjugated with glycol chitosan via a dehydrated 3,3-dithiodipropionic acid linker that was cleaved in 10 mM GSH [52].

GSH-triggered GTs are commonly prepared in two major ways: (1) attaching a thiol moiety to the terminal end of a polymer and self-crosslinking them in an appropriate solvent and (2) attaching a nucleic acid-containing terminal thiol moiety to the surface of metal nanoparticles, such as gold or silver, which have high affinity for thiols.

Reducible disulfide-crosslinked cationic polymer derivatives are largely obtained by two procedures, i.e., by introducing disulfide bond-containing crosslinkers or by a pre-thiolation strategy [100,101]. In either case, low molecular weight polymers, either linear or branched, were mostly used in order to reduce cytotoxicity [102]. Recently, Wen et al. introduced a new reducible supramolecular cyclodextrin polyrotaxanes-based copolymer (SS-PR), which consisted of α-cyclodextrin-based PR and a disulfide-linked poly(2-dimethylaminoethyl methacrylate) (pDM) block prepared via in situ atom transfer radical polymerization (ATRP) of 2-(dimethylamino)ethyl methacrylate (DMAEMA), for delivering pDNA. Introducing a disulfide linker between the two copolymers enabled them to dissociate in the presence of intercellular GSH, leading to efficient gene release, and the plasmid DNA showed higher accumulation around the nucleus, which resulted due to the GSH-mediated gene release in HeLa cells [103].

Metallic nanoparticles such as gold or silver have a strong affinity toward thiol moieties and thus became the platform for developing metal-based GT. Thiolated siRNA can directly bind to the surface of gold nanoparticles via thiol linkages [104] and can also form polyplexes with a gold-polymer hybrid via electrostatic interaction in a single layer or layer-by-layer method [105,106]. Kong et al. developed multimerized siRNA crosslinked by gold nanoparticles using both these techniques. In this study, they attached the thiolated single-stranded sense and antisense strands to gold nanoparticles, then formed dimerized gold clusters, and finally prepared polyplexes with linear PEI. This nanoparticles had a size of 319 nm and a surface charge density of +13.2 ± 3.4 mV, and reductive enzyme-mediated gene release was obtained at 300 µM GSH with complete protection against RNase enzymes [107].

Another strategy involves the chemical self-crosslinking and multimerization of biologically active siRNA structures through cleavable disulfide linkages. Hyeung et al. demonstrated that multimeric siRNA can be synthesized by dimerizing self-crosslinked single-stranded sense and antisense strands using a GSH-cleavable maleimide containing linker and then creating nanosize particles using a cationic polymer, such as linear PEI [108,109]. This multimerized siRNA provided a unique opportunity to target multigenes and demonstrated effective gene release in a reductive environment. GSH-dependent enzyme-triggered gene transfer is a common intracellular trigger and requires incorporation of a disulfide bond into the nanoparticle for intracellular gene release.

### 3.2. Light-Mediated Gene Release (L-TGR) 

Photo stimuli-responsive gene carriers are considered to be more efficient than conventional gene carriers in terms of their efficacy as therapeutics and reduction in side effects because light-mediated gene delivery, an “on-demand” gene release technique, requires a photosensitizer that either induces cleavage or emits heat energy upon irradiation with a light source (NIR or ultraviolet (UV)). Using this technique, off-target gene expression can be avoided, and the therapeutic gene silencing effect or plasmid expression in the tumor tissue becomes more efficient. At an intracellular level, the major obstruction to nucleic acid release in the cytosol is the membrane barrier of the endocytic vesicles. Currently, novel techniques such as photochemical internalization (PCI) are used for releasing nucleic acid cargo from the endocytic vesicle to the cytosol [110]. Designing a GT based on the PCI technique can be achieved by incorporating a photocleavable linker into the polymer, which will release the nucleic acid upon irradiation with a NIR laser or UV/Visible light. Another way of performing PCI is to deliver the nucleic acid along with a photothermal agent, which promotes endosomal escape (Figure 2). 

#### 3.2.1. Photothermally Triggered Gene Release

NIR light ranges from 700 to 2500 nm in wavelength and can penetrate through body tissue such as blood and skin more efficiently than visible light, although water and lipids show increased absorption in the NIR range above 950 nm [111]. Hence, the NIR window between 600 and 950 nm is optimal for in vivo imaging and for photothermally mediated therapy. After absorbing NIR light, the subatomic particles in the nanoparticles are excited from the ground state to the excited state, and then, upon returning back to the ground state through non-radiative decay channels, they emit kinetic energy, leading to the production of heat energy [112]. However, the major concern in developing GT based on this method is that high laser power (W/cm^2^) or increased photothermal therapy (PTT) agent concentration will cause major safety issues, such as cytotoxicity, morbidity, and lack of patient tolerance.

**Figure 5:**
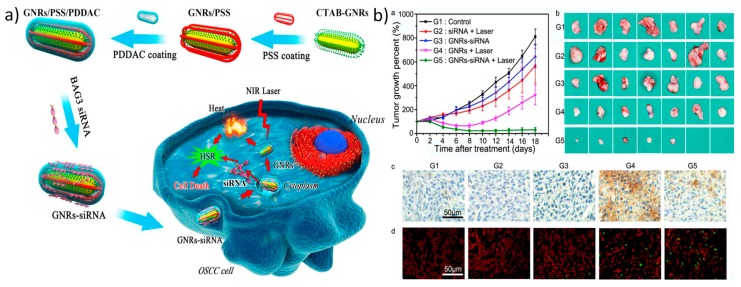
Photothermally mediated gene delivery using metal-based gene transporters: (**a**) Schematic illustration of the design of GNRs-siRNA in an improved PTT platform; (**b**) GNRs-siRNA inhibited tumor growth in a xenograft model after irradiation with an 810 nm laser: (**a**,**b**) Mean tumor growth percent of different treatment in the xenograft model; (**c**) Immunochemistry of BAG3 expression and (**d**) TUNEL assay showing apoptotic cells in tumors after 24 h. Reproduced with permission from [117]. Biomaterials, Elsevier 2017.

The most popular metal-based nanomaterials for photothermally mediated gene delivery are gold nanoparticles, such as gold nanostars, nanorods, and nanocages, which, based on their morphological structure, shift their surface plasmon resonance from the visible range to the NIR range. Their affinity toward thiol groups provides the opportunity for thiolated nucleic acids, such as siRNA or oligonucleotides, to bind to and be released from the particles once they are irradiated with a NIR laser [113,114]. Gold nanoparticles either can be employed without any modification or can be coated with cationic polymers for efficient gene release/delivery [115,116]. Recently, Wang et al. developed gold nanorods coated with positively charged poly(diallyldimethylammonium chloride) (PDDAC) and negatively charged poly (sodium 4-styrene sulfonate) (PSS), which are used for the delivery of BAG3-targeting siRNA. A major aspect of this study to be taken into consideration is that they used a low power laser, which prevents abnormal circumstances, such as protein degradation or apoptosis initiation, in the cells (Figure 5) [117].

In addition to delivering nucleic acids such as siRNA, miRNA or plasmids using photothermal methods, Jung et al. presented an interesting method of enhancing the delivery of oncolytic adenovirus (Ad)-expressing vascular endothelial growth factor (VEGF) promoter-targeted artificial transcriptional repressor zinc-finger protein to head and neck cancer cells by inducing mild hyperthermia using gold nanorods in vitro and in vivo which leads to their cellular uptake [118]. 

**Figure 6:**
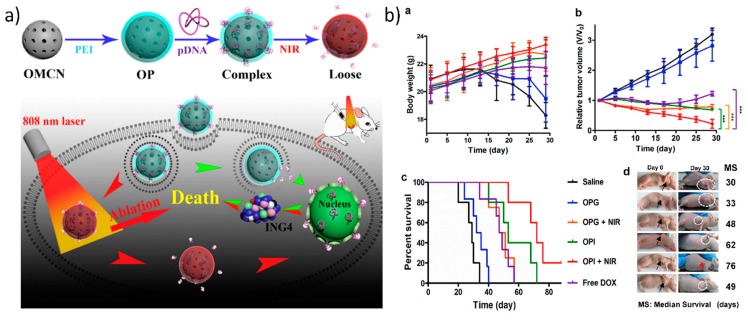
Photothermally mediated gene delivery using carbon-based gene transporters: (**a**) Illustration of the synthesis of and photothermal combined gene therapy achieved by polyethylenimine (PEI)-grafted oxidized mesoporous carbon nanospheres (OMCN), (**b**) the application of which resulted in tumor growth inhibition after irradiation, which led to release of the plasmid ING4 (pING4) complexed with the PEI–grafted OMCN and its expression in breast cancer tumor-bearing nude mice: (**a**,**b**) mean body weight and relative tumor volume of treated mice; (**c**,**d**) survival curves and tumor images of mice on 30th day post injection with different treatments. Reproduced with permission from [120]. Biomaterials, Elsevier 2017.

Additionally, utilizing carbon-based nanomaterials as a photothermally mediated gene delivery approach is attractive due to the presence of sp^2^-hybridized carbon atoms, which are responsible for the photothermal heat conversion of NIR radiation. For photothermally mediated gene delivery, two widely used carbon-based GT with tremendous photothermal properties are one-dimensional carbon nanotubes and two-dimensional reduced graphene oxide [49,62,119]. Recently, three-dimensional carbon nanospheres have also emerged because of their photothermal properties. Meng et al. developed PEI-grafted mesoporous carbon nanospheres that destabilized the endosomal/lysosomal vesicles in cells which triggered the release of plasmid ING4 into the cytoplasm upon irradiation with 808 nm laser at 1 W/cm^2^. This then led to apoptosis/cell death caused by the ING4 protein and to the generation of thermal heat by the carbon nanoparticles, as shown in Figure 6 [120]. However, the major drawback of employing graphene or any other carbon-based nanomaterial is that they have long-term toxicity in the body. Therefore, it is necessary to design carbon-based gene carriers in such a way that they are degraded quite easily by the body. Kim et al. showed systematically that graphene oxide conjugated via a thiol bond to thiolated PEI and PEG was degraded by macrophage cells. Here, the graphene oxide was cleared from the polymer coating, was then exocytosed after the photothermal endosomal disruption, and was later degraded by peroxidase enzymes in the macrophages [121].

In addition to graphene, other two-dimensional nanomaterials include exfoliated transition metal dichalcogenides, such as molybdenum disulfide (MoS_2_), tungsten sulfide (WS_2_), molybdenum diselenide (MoSe_2_), and tungsten diselenide (WSe_2_), which share similar properties to graphene. Compared to graphene, these transition metals possess very low cytotoxicity toward cancer cells, even at higher concentrations [122]. Kou et al. reported that nanocomposites synthesized by conjugating PEG and PEI-lipoic acid to MoS_2_ via disulfide linkages had good biocompatibility with reduced cytotoxicity, as well as high gene-carrying ability without serum interference, thus resulting in high gene release and transfection [123]. Furthermore, Jinhwan et al. showed that this nanocomposite was released from the endosome/lysosomal vesicles after photothermal irradiation and then released the genes only after the disulfide bond was reduced by the GSH in the cytoplasm [124]. 

In photothermally mediated gene delivery, developing a low cytotoxicity and target-specific gene transporter is very important. For instance, carbon-based GT exert a toxic effect on cancer cells as well as healthy cells by inducing oxidative stress, and studies have been shown that one of the factors causing this effect is the surface modification of the carbon-based GT [125,126,127]. Therefore, surface modification methods should be biocompatible, and cancer-specific targeting ligands should be incorporated. 

Photothermal agents are composed of metals, carbon materials, polymers, and dyes that have low quantum yields to produce elevated heat in cells. Xue et al. showed that chitosan-coated Prussian blue/iron oxide nanoparticles had strong gene binding capacity as well as photothermal heat conversion, and they were taken up by cells in response to a magnetic field and released DNA into the cytoplasm after photothermal irradiation [128]. One important aspect that should be taken into consideration is that delivering PTT GT intravenously requires a ligand that targets a specific organ site. The therapeutic efficacy of the GT also depends on the concentration of nanoparticles entering the tumor site. Hence, a targeting ligand has to be conjugated to the surface of the particles in order to achieve high antitumor efficacy.

#### 3.2.2. Photochemical Internalization (PCI)-Triggered Gene Release

The light-induced rupture of endocytic membranes is triggered by the use of a photosensitizer localized in the cell membrane and, upon irradiation, creates reactive oxygen species (ROS) that destroy the cell membrane and induce cell apoptosis. In addition, it destabilizes the endocytic vesicles in cells, allowing the nanoparticle cargo to escape the lysosomal degradation and the photosensitizer (PS) to bind to the cell membrane surface and to be internalized along with the cargo, which later gets released after irradiating the cell with a light source [129]. Furthermore, a PSs should be amphiphilic because they should not penetrate or intercalate with the cellular membranes and should be present in the endosome vesicle surface while internalizing the gene cargo [9,130,131]. In the case of gene delivery, the short range of action and lifetime of ROS production can avoid damaging effects, and therefore, they can just release their cargo after disrupting the endocytic vesicles [132]. Selbo et al. performed a study that focused on delivering three components, i.e., doxorubicin, the ribosome-inactivating protein gelonin, and the E1/E3-deleted adenovirus serotype 5 (Ad5) vector Ad5CMV-lacZ, into the multidrug resistant (MDR) uterine fibrosarcoma cell line MES-SA/Dx5 using the photosensitizer disulfonated meso-tetraphenylporphine (TPPS2a). The increase in light exposure resulted in the decrease in the MDR property of the cells, suggesting an increase in adenovirus transduction effected by P-gp suppression [133]. Similarly, Oliveira et al. utilized the same photosensitizer to deliver EGFR siRNA in a human epidermoid carcinoma cell line (A431 cell line) and found a 10-fold increase in the knockdown of EGFR protein expression after light irradiation at 375–450 nm with 13 mW/cm^2^ power [134].

Until now, the PCI technique has been employed only to induce viral or naked gene escape from the endosomal vesicles, although it is inevitable that non-viral gene transporters will be used for in vivo gene delivery. Zamora et al. delivered the tumor suppressor gene phosphatase and tensin homolog (PTEN) and the cytosine deaminase (CD) pro-drug activating gene into photosensitizer (AlPcS_2a_)-treated U87 and U251 glioma cell monolayers and multicell tumor spheroids using polyamine protamine sulfate/Eosin 5-isocitrate conjugated polyplexes with acid degradable monomers polymerized into shelled nanoparticles, followed by a 670 nm laser at 5 mW/cm^2^ [135,136]. Similarly, PEI-coated poly(d,l-lactide-*co*-glycolide) (PLGA)/DNA complexes, cationic dextran nanogels, and polyamidoamine (PAMAM) dendrimers have been used as GT for delivering genes via, the PCI technique [137,138,139].

### 3.3. Ultrasound-Mediated Gene Release (US-TGR)

Although microbubbles (MBs) have been the main contrast agent for ultrasound imaging for decades, they are currently employed as drug and gene delivery agents as well. Recent studies showed that microbubbles can be synthesized using different formulations, such as liposomes loaded with a liquid/gas mixture or phase shift liquid droplets that can convert to a gas bubble upon exposure to ultrasound. Ultrasound-mediated gene delivery or sonoporation is achieved by the collapse of these microbubbles after the application of cyclic sound pressure in a particular frequency range (>20 kHz), which leads to a change in permeability of the cell plasma membrane, finally releasing the cargo in the cytoplasm.

#### 3.3.1. US Microbubble-Triggered Gene Release

Gene transfer using microbubbles is achieved using a gas or phase shift liquid encompassed in biocompatible lipid shells, proteins or polymers, and their physical structure makes them suitable gene carriers because prolongation of the half-life of the therapeutic substances is achieved. The triggered release at a region of interest via focused ultrasound (FUS) sonication is also possible, and microbubble-assisted gene transporter delivery has more therapeutic efficacy than delivery mediated by cationic polymers. The shell of the MBs provides the conjugation site for the targeting markers and allows them to be selectively aggregated to the target cells. Ching-Hsiang et al. delivered the red firefly luciferase gene (pFLuc) across the blood-brain barrier (BBB) to C6 glioma cells using folate-conjugated lipids consisting of Dipalmitoylphosphatidylcholine (DPPC), 1,2-dipalmitoyl-3-trimethylammonium-propane (DPTAP), and DSPE-PEG_2000_ loaded with perfluoropropane (C_3_F_8_) gas [140]. The cavitation and radiation force generated by the FUS and MBs provided locally and temporally increased permeability of the BBB and allowed particles smaller than 187 nm to transvascularly enter the brain tissue. 

To increase the transfer of nucleic acids into the target site via ultrasound, microbubbles require high ultrasound energy, although it also affects the integrity of the cell membranes and causes cell death [141]. However, this can be avoided by using low-frequency and low-energy ultrasound with commercially available microbubbles, a strategy which increased the expression of tumor suppressor gene P53 in PC-3 prostate cancer cells and suppressed the expression of long noncoding activated-RNA by transforming growth factor-β (TGF-β; lncRNA-ATB) in hepatocellular carcinoma (HCC) cells [142,143].

Ultrasound-assisted gene delivery provides a unique opportunity to assess the therapeutic efficacy of gene/MBs by imaging the tumor with real time monitoring [144]. Wang et al. used an anti-apoptotic X-linked inhibitor of apoptosis protein (XIAP) siRNA encapsulated within a cationic diblock copolymer micelle and later coated over the surface of the MBs for theranostic applications in human cervical cancer xenograft models [145].

The main role of the US microbubbles is in permeabilizing the membranes of cells/tissue and allowing cargo to enter the cells or tissue site [146]. However, for effective gene transfection, the surface charge of cationic polymers should also be considered as an important factor in US-mediated gene delivery [147,148]. Nucleic acid delivery by a microbubble-assisted gene transporter is comparatively superior to delivery by cationic polymers in terms of therapeutic efficacy. Florinas et al. coated microbubbles with arginine-grafted bio-reducible poly(disulfide amine) (ABP)-VEGF siRNA polyplexes and showed that the US-mediated delivery of these polyplexes effected a greater reduction in tumor volume than was observed in the A2780 human ovarian cancer xenograft model treated only with polyplexes [149]. 

The major limitation of this US microbubble gene transfer approach is that it cannot pass through the vessel wall of the tumor tissues, although it can assist with the release of the gene and therapeutic drugs from the carrier near the tumor environment [150,151,152]. Another disadvantage of using gas phase perfluorocarbon (PFC)-containing microbubble is that they have poor resistance toward any distortions, thus leading to a short circulation time in vivo. Gao et al. loaded liquid PFC into plasmid DNA-cationic nanodroplets containing the polymers C_11_F_17_-poly *N*-[*N*′-(2-aminoethyl)] aspartamide [C_11_F_17_-PAsp (DET)] to obtain enhanced stabilization, biocompatibility, transgene expression, and US contrast effect [153]. 

#### 3.3.2. US Nanobubble-Mediated Gene Transfer

The major disadvantages of microscale bubbles can be avoided by using nanobubbles, which are much better suited for US-mediated targeted gene delivery [154,155]. Nucleic acids can be delivered with nanobubbles by several methods. Horie et al. delivered tumor necrosis factor-α (TNF-α) encoded plasmid with lipid-based nanobubbles via intratumoral injection into a murine breast carcinoma (EMT6) tumor model. The expression of the P53 tumor suppressor gene and apoptotic caspase 3 gene was proportionally increased according to the increase in TNF-α expression affected by the treatment with the US nanobubbles [156].

To achieve a synergistic effect from both drugs and genes in cancer cells, US nanobubbles can be used to deliver drugs and genes using the hetero-assembly of cationic polymer micelles containing therapeutic gene and hydrophobic drug-loaded liposomes containing a US gas agent [50]. Coating the cationic micelles with genes over the liposomes containing the US gas agent, such as perfluorocarbon, improved the bioavailability of the gene in tumor tissue and increased its therapeutic efficacy [157]. Yin et al. coated the gas-cored liposomes with cationic micelles prepared from poly(ethylene glycol)-*b*-poly (benzyloxycarbonyl-l-lysine) copolymer (mPEG-*b*-PCBLLys) complexed with siRNA. The nanobubbles released sirtuin 2 (SIRT2) siRNA from the cationic micelles, resulting in a drastic reduction in tumor volume and an improvement in survival in the US nanobubble-treated C6 glioma xenograft tumor model [50]. 

Xie et al. conjugated penetratin as a cell penetrating peptide to c-Myc siRNA for specificity and then entrapped the conjugates into ephrin mimetic peptide modified nanobubbles. Once the nanobubbles bound to the target site of EphA2 receptors on cancer cells, ultrasound stimulation triggered the release of the CPP-siRNA into specific tumor cells only [158]. 

### 3.4. Magnetic Nanoparticle Mediated Gene Transfer (M-TGR) 

Magnetic field-mediated gene transfer is achieved by applying an external magnetic field to target cells or a tissue site with nucleic acids that are bound to magnetic nanoparticles (MNPs) in vitro and in vivo [79,159,160]. Here, the magnetic field does not alter the cellular uptake mechanism but instead leads the polymer-coated magnetic particles to sediment over the cell surface and enter the cells by normal endocytosis, thus avoiding the proton sponge effect [161,162]. Lo et al. formulated polymer-coated magnetic nanoparticles by chemically conjugating chondroitin sulfate (CS) to PEI-decorated SPION for the delivery of miR-128 in U87 xenograft-bearing mice by in vivo magneto-induced uptake [51]. The magnetic field applied near the tumor site improved the particle accumulation on the cell surface, and the CS helped with CD44-based cellular uptake, thus improving the therapeutic activity of miR-128 in the tumor [163,164]. The enhanced sedimentation of the magnetic particles results from applying the magnetic field over the cell surface. Pavlov et al. showed a rapid sedimentation rate and improved contact of layer-by-layer microcapsules consisting of PEI, dextran and iron oxide nanoparticles loaded with CaCO_3_/luciferase DNA. Luciferase enzyme delivery with these capsules led to an increase in gene expression as well as higher enzyme activity inside cells [165].

Magnetic nanoparticles have been used as hyperthermia agents to kill cancer cells because the magnetic nanoparticles with higher specific absorption rate (SAR) values have the ability to generate heat in the presence of an alternative magnetic field (AMF) [166,167]. In addition, a recent study showed that along with delivering a therapeutic gene via magnetofection, they sensitized cells and then induced hyperthermia to completely destroy the cells [168]. Interestingly, Yin et al. delivered lethal-7a miRNA (let-7a) using PEI-coated 2,3-dimercaptosuccinic acid (DMSA)-stabilized zinc-doped iron oxide nanoparticles (ZnFe_2_O_4_) by applying a magnetic field over U87-EGFRvIII GBM cells and later applied an alternative magnetic field (AMF) to generate hyperthermia [169]. This can be referred to as the “stop and kill them method” because let-7a miRNA is a tumor suppressor that inhibits the malignancy of cancer cells by downregulating the downstream molecules in the BRCA family and other heat shock proteins, such as HSP 70 and HSP90. Sensitization of the cells by let7a miRNA induces rapid cell death by hyperthermia.

It should be noted that the degradation of polymer-coated magnetic particles is necessary because cytotoxicity and poor transgene expression can occur [170,171]. However, it has been suggested that the loading of magnetic nanoparticles into a biodegradable lipid or polymer can prevent these consequences [172,173,174]. Shang et al. and Hu et al. concealed cationic lipids and plasmid DNA inside the core of a liposome along with iron oxide nanoparticles to produce a surfactant-free, biocompatible gene transporter, the cellular uptake of which increased 30–40-fold due to magnetic field guidance [175].

The versatility of iron oxide nanoparticles comes from their ability to combine with other metal ions and form a multimodal entity with synergistic optical and electronic properties [176]. Shi et al. developed Au-Fe2O4 dumbbell shape nanoparticles to enhance magnetofection as well as to improve the capacity of micro-optical coherence tomography (μOCT) to track the particles [177]. 

## 4. Challenges Associated with and Future Directions of Trigger-Responsive Gene Transporters

In particular, making GT safe and less toxic for preclinical and clinical trials is the most important factor to be taken into consideration. Although carbon based GTs such as graphene and carbon nanotubes have shown great efficacy in photothermal mediated gene delivery, they possess genotoxicity and elicit inflammatory responses [178,179,180]. In L-TGR, it is quite important that the cells are treated with the optimum amount of GTs that provides heat or ROS enough to escape from the endosome/lysosome. Mild hyperthermia can suffice enough to break the endosome/lysosomal vesicle and also intercellular proteins that become denatured at this temperature (42 °C) can also renature again [118]. Another roadblock for L-TGR is that it requires high accumulation of GTs in the tumor site in order to elevate the temperature using laser irradiation. GTs are non-toxic up to a particular range and beyond that quite toxic to the cells. And also they need to surpass the non-specific tissue accumulation and become concentrated more in the tumor site. Therefore, future design of L-TGR based GTs should also consider tumor accumulation as one of the important criteria. Recently, cancer cell membrane (CCM) vesicle based nanoparticles have shown tumor specific targeting as well as higher accumulation [181]. CCM can accommodate GTs like PLGA with high payload of both hydrophobic and hydrophilic cargos [182]. Effective function of triggers also depends on the size of nanoparticle, especially in M-TGR based GTs. Here, the SPION size has to be >100 nm and carry a tumor targeting ligand in order to obtain magnetic mediated targeting as well as accumulation in the tumor tissues [183]. Rather than developing GTs responsive to individual triggers, it will be better to combine multiple triggers in order to obtain high gene transfection efficiency with low cytotoxicity. For example, an internal GSH trigger combining with one of these triggers such as light, magnetic field or ultrasound will minimize the injection dose of GT, increase the transgene expression along with unique opportunities to visualize the therapeutic outcome in real time. 

In the future, focus should be on utilizing dual modality gene transporters in order to improve the therapeutic efficacy of the gene. Huang et al. recently fabricated a multi-theranostic nanobubble system by synthesizing nanobubbles using mesoporous silica SPION shell nanoparticles loaded with perfluoropentane. They act as Magnetic resonance imaging (MRI)/US dual-modality contrast agent and cause FUS-induced BBB disruption [184]. This multimodal nanoparticle imaging system has the potential to be used along with gene carriers to deliver therapeutic genes across the BBB for the treatment of glioma cancer. Similarly, Liu et al. synthesized gold nanorods (GNRs) loaded into nanobubbles, which acted as an ultrasound agent and had photothermal properties due to the GNRs [185]. For example, This theranostic nanosystem can enhance the availability of the GNRs with nucleic acids at the tumor site via ultrasound-mediated release and induce photothermally mediated gene release in the cancer cells.

## 5. Conclusions

Immense progress in the field of trigger-responsive gene carriers has led to the development of novel therapeutic strategies to cure or prevent cancers. With a greater understanding of the physiological differences between normal and diseased tissues and advances in material design, there is an opportunity to develop gene transporter systems for target-specific gene delivery that will respond to local stimuli. This review summarized the role of enzyme-, light-, ultrasound-, and magnetic field-responsive gene carriers in targeted gene delivery. In addition to targeted delivery, some of these triggered-gene transporters have the potential to be applied in other non-cancerous cells, such as immune cells or stem cells that can be used indirectly for the treatment of cancers. Perhaps the focus should now shift toward clinically acceptable systems that are more sensitive to discrete variations in specific stimuli. The ‘on demand’ release of a gene requires external as well as internal stimuli, and the design of nanoparticles should be a point of emphasis in developing multi-stimuli-responsive gene carriers. Furthermore, this strategy demands drugs to be used along with genes to produce a synergistic effect in cancer cells.

## Figures and Tables

**Table 1:** List of trigger-based gene transporters for anticancer gene therapy.

Trigger	Gene Transporter	Gene	Trigger’s Outcome	Reference
**Enzyme-triggered gene release (Enz-TGR)**				
**Glutathione-dependent enzyme-triggered gene release**	Glycol chitosan conjugated to low molecular weight polyethylenimine (PEI) via a disulfide bond (GCS-ss-PEI)	GFP plasmid DNA	Low cytotoxicity, higher transgene expression, GSH responsive.	[52]
	Cationic folic acid and camptothecin conjugated four-arm PEG micelle	Tumor necrosis factor-α (TNFα)-encoded plasmid	GSH-mediated TNFα plasmid DNA release, increased survival rate, reduced tumor metastasis, suppressed 4T1 tumor growth.	[53]
	Fluorinated bioreducible *N*,*N*-dimethyldipropylenetriamine polymer	Luciferase silencing RNA (LucsiRNA)	Low cytotoxicity, high gene silencing efficiency, GSH-mediated siRNA release, high cell internalization and buffering capacity.	[54]
	Surface charge-switchable folate modified co-delivery system and tumor-targeting polypeptide (FK)/PEG-2,3-dimethylmaleic anhydride-modified-PLL	P53-expressing plasmid	GSH-mediated release of proapoptotic peptide C-KLA (TPP) and p53 plasmid, high particle accumulation in tumor.	[55]
**Protease-triggered gene release**	MMP2-sensitive self-assembling copolymer, polyethylene glycol-peptide-polyethylenimine-1,2-dioleoyl-sn-glycero-3-phosphoethanolamine (PEG-pp-PEI-PE)	Anti-survivin siRNA	Successful cancer cell-selective co-delivery of siRNA and paclitaxel, higher cellular uptake and exposure of hidden PEI by MMP2 cleavage.	[56]
	MMP2-sensitive peptide-CPP arginine (R9) peptide conjugated in between PEG and poly(ε-caprolactone) (PCL) in a micelle	Anti-Plk1 siRNA	Effective gene silencing, selective uptake of micelle in MMP2-overexpressing cancer cells.	[57]
	MMP2-cleavable substrate peptide conjugated cationic β-cyclodextrin-polyethylenimine conjugates (En-CNP)	microRNA miR-34a	High transfection of miR-34a inhibited 4T1 tumor growth. Increase in particle accumulation in tumor along with reduced accumulation in the liver.	[58]
	siRNA complexed cationic liposome consisting of PEG_2000_-peptidyl lipids with peptidyl moieties sensitive to MMP2	Anti-luciferase siRNA	Increase in knockdown of luciferase expression in the stable luciferase-expressing cells MCF-7-luc and HT1080-luc.	[59]
**Light-triggered gene release (L-TGR)**				
**Photothermally triggered gene release**	Gold nanorod-embedded large-pore mesoporous organosilica (GNR@LPMO) nanospheres	PLK1 siRNA	Effective gene release by photothermal irradiation, released PLK1 siRNA lowered PLK1 gene expression, induced early apoptosis, reduced tumor volume.	[60]
	Chitosan-functionalized copper sulfide nanoparticles (CuS@CS NPs )	Luciferase plasmid	Increase in luciferase expression after irradiation compared with PEI transfected cells.	[61]
	Single-walled carbon nanotube (SWCNT) wrapped with poly(ethylenimine)-cholesterol (PCS)	TP53 plasmid	Increase in TP53 expression, three-fold reduction in tumor volume compared to non-irradiated tumor.	[62]
	SWCNT conjugated PEI	hTERT siRNA	hTERT expression reduced in PC-3 tumor, resulted in decrease in tumor growth after Near infrared (NIR) irradiation.	[63]
**Photochemical internalization/photosensitizer-triggered gene release**	Photosensitizer (TatU1A-dye)-labeled cell penetrating peptide (TAT) conjugated with RNA binding protein	EGFP shRNA	EGFP silencing efficiency after irradiation is 80% in the stable EGFP-expressing CHO cell line compared to non-irradiated cells.	[64]
	Dendrimer phthalocyanine micelle coated over gold nanorods	Venus, yellow fluorescent protein (YFP)-expressing plasmid	YFP expression increased 5 times more in HeLa tumor than in non-irradiated tumor	[65]
	Pheophorbide-a (PhA)-conjugated chondroitin sulfate complexed PEI polyplex	EGFR-shRNA	HCT116 tumor growth drastically reduced with an increase in EGFR gene silencing after irradiation.	[66]
	Pegylated oligoethylenimine (OEI) conjugated to TPECM via an aminoacrylate (AA) linker	EGFP plasmid	After irradiation, enhanced gene expression in HeLa cells with higher cell viability.	[67]
**Ultrasound-triggered gene release (US-TGR)**				
**Microbubble ultrasound-triggered gene release**	Lipid-based microbubble conjugated with polystyrene nanospheres and mRNA lipoplexes.	Luciferase mRNA	Increase in diffusion of mRNA lipoplexes into the cells through the membrane pores caused by cavitation microbubbles upon US irradiation.	[68]
	PLGA/PEG nanoparticles delivered along with microbubble	miR-122 microRNA	Increase in the accumulation of miR-122 after US irradiation.	[69,70]
	PEGylated siRNA/lipid complexes conjugated over lipid-based microbubble via biotin-avidin conjugate.	Luciferase siRNA	Decrease in luciferase expression in HUH7eGFPLuc cells after US irradiation.	[71]
	TAT peptide-labeled PEG-modified liposomes (TAT-PEG liposomes) along with bubble liposomes	Luciferase plasmid	Increase in luciferase expression in HeLa cells upon US exposure.	[72]
**Nanobubble ultrasound triggered gene release**	DOX-PLGA/PEI/P-gp shRNA nanobubbles	P-gp shRNA	Decrease in P-gp expression, and increased in DOX-mediated cell toxicity in MCF-7/ADR after US irradiation.	[73]
	Cell penetrating peptide-siRNA conjugate loaded in liposome nanobubbles	Anti-c-myc siRNA	Inhibition of HT-1080 tumor due to the silencing of c-Myc by siRNA delivered through US exposure.	[74]
	siRNA/cationic liposome conjugated with glypican-3 (GPC3) antibody via biotin-avidin nanobubble (siRNA TNB) complexes	Neuroepithelial transforming protein 1 (NET-1) siRNA	Substantial increase in gene silencing efficiency after exposing the nanoparticles to low-frequency US.	[75]
	Mannosylated PEG nanobubble lipoplexes	Nf-KappaB decoy oligonucleotide (NKBDO)	Increase in transfection of oligonucleotide due to the US exposure, reduced solid tumor growth.	[76]
**Magnetic-triggered gene release (M-TGR)**				
	PAMAM dendrimer-coated magnetic nanoparticles (DcMNP)	CpG oligonucleotide	Higher cell apoptosis in MDA-MB-231 and SKBR3 cells.	[77]
	Disulfide PEI-coated SPION (PSPIO)	pcDNA3.Luciferase plasmid DNA	High gene transfection efficiency in the presence of serum after magnetic field exposure.	[78]
	Chitosan magnetic nanoparticles	TNF-related apoptosis-inducing ligand (TRAIL)-expressing plasmid	Increase in TRAIL gene expression after magnetofection caused apoptosis in cancer cells.	[79]
	PEI-modified Fe_3_O_4_ nanoparticle	pACTERT-TRAIL plasmid	Increase in apoptosis induced in SACC-83 cells and Tca83 cells by TRAIL gene expression after magnetic field application.	[80,81]

## Abbreviations

The following abbreviations are used in this manuscript:

AMF	Alternative magnetic field
ATRP	Atom transfer radical polymerization
BBB	Blood Brain Barrier
CCM	Cancer cell membrane
CD	Cytosine deaminase
CPP	Cell penetrating peptide
CPT	Camptothecin
DA	2,3-Dimethylmaleic anhydride
DET	Diethylenetriamine
DMAEMA	2-(Dimethylamino)ethyl methacrylate
DMSA	2,3-Dimercaptosuccinic acid
DNA	Deoxyribonucleic acid
DOSPA	2,3-Dioleyloxy-*N*-[2-spermine carboxamide] ethyl-*N*,*N*-dimethyl-1-propanammonium trifluoroacetate
DOTAP	1,2-Dioleoyl-3-trimethylammonium-propane
DOTMA	*N*-[1-(2,3-dioleoyloxy)propel]-*N*,*N*,*N*-trimethylammonium
DOX	Doxorubicin
DPPC	Dipalmitoylphosphatidylcholine
DPTAP	1,2-dipalmitoyl-3-trimethylammonium-propane
DSPE-PEG 2000	1,2-distearoyl-sn-glycero-3-phosphoethanolamine-*N*-[amino(polyethylene glycol)-2000]
EGFR	Epidermal growth factor receptor
Enz-TGR	Enzyme triggered gene release
GCS	Glycol chitosan
GFP	Green fluorescent protein
GNR	Gold Nanorod
GSH	Glutathione
GSSG	Glutathione disulfide
GT(s)	Gene transporter(s)
HIV	Human immunodeficiency virus
L-TGR	Light triggered gene release
MB	Microbubble
MDR	Multidrug resistant
miRNA	MicroRNA
MMP2	Matrix metalloproteinase-2
MRI	Magnetic Resonance Imaging
mRNA	Messenger RNA
M-TGR	Magnetic field triggered gene release
NET	Neuroepithelial transforming protein 1
NIR	Near infrared
NKBDO	Nf-KappaB decoy oligonucleotide
OMCN	Oxidized mesoporous carbon nanospheres
OEI	Oligo ethylenimine
PABC	*p*-Amino benzyloxy carboxyl
PAGA	Poly(aminolated glycidyl methacrylate)
PAMAM	Polyamidoamine
PCI	Photochemical internalization
PCL	Poly(ε-caprolactone)
PDDAC	Poly(diallyl dimethyl ammonium chloride)
PDGFR-α	Platelet-derived growth factor receptor alpha
PDPA	Poly(2-(diisopropyl amino) ethyl methacrylate)
PE	Phosphoethanolamine
PEG	Polyethylene glycol
PEI	Polyethylenimine
PF14	PepFect14
PFC	Perfluorocarbon
PhA	Pheophorbide-a
PLGA	Poly(lactic- *co*-glycolic) acid
PLL	Polylysine
PSS	Poly(sodium 4-styrene sulfonate)
PTEN	Phosphatase and tensin homolog
PTT	Photothermal therapy
RNA	Ribonucleic acid
ROS	Reactive oxygen species
SAR	Specific absorption rate
siRNA	Silencing RNA
SIRT2	Sirtuin 2
SPION	Superparamagnetic iron oxide nanoparticle
SWCNT	Single-walled carbon nanotube
TAT	HIV transactivator
TNF-α	Tumor necrosis factor-alpha
TPP	Triphenylphosphonium
TRAIL	TNF-related apoptosis-inducing ligand
US-TGR	Ultrasound triggered gene release
VEGFR-2	Vascular endothelial growth factor receptor-2
XIAP	X-linked inhibitor of apoptosis protein
μOCT	Micro-Optical Coherence Tomography
